# Interrelationships of Fiber-Associated Anaerobic Fungi and Bacterial Communities in the Rumen of Bloated Cattle Grazing Alfalfa

**DOI:** 10.3390/microorganisms8101543

**Published:** 2020-10-07

**Authors:** Elnaz Azad, Kelsey B. Fehr, Hooman Derakhshani, Robert Forster, Surya Acharya, Ehsan Khafipour, Emma McGeough, Tim A. McAllister

**Affiliations:** 1 Department of Animal Science, University of Manitoba, Winnipeg, MB R3T 2N2, Canada; azade@myumanitoba.ca (E.A.); fehrk345@myumanitoba.ca (K.B.F.); Ehsan_Khafipour@diamondv.com (E.K.); Emma.Mcgeough@umanitoba.ca (E.M.); 2 Department of Medicine, McMaster University, Hamilton, ON L8N 3Z5, Canada; derakhsh@mcmaster.ca; 3 Lethbridge Research and Development Centre, Agriculture and Agri-Food Canada, Lethbridge, AB T1J 4B1, Canada; forster.micro@gmail.com (R.F.); surya.acharya@canada.ca (S.A.); 4 Diamond V, Cedar Rapids, IA 52404, USA

**Keywords:** rumen fungi, bloat, sainfoin, alfalfa, cattle grazing

## Abstract

Frothy bloat is major digestive disorder of cattle grazing alfalfa pastures. Among the many factors identified to contribute to the development of frothy bloat, the disruption of rumen microbiota appears to be of central importance. Anaerobic rumen fungi (ARF) play an important role in sequential breakdown and fermentation of plant polysaccharides and promote the physical disruption of plant cell walls. In the present study, we investigated the dynamics of ARF during the development of alfalfa-induced frothy bloat and in response to bloat preventive treatments. By sequencing the internal transcribed spacer (ITS1) region of metagenomic DNA from the solid fraction of rumen contents, we were able to identify eight distinct genera of ARF, including *Neocallimastix*, *Caecomyces*, *Orpinomyces*, *Piromyces*, *Cyllamyces*, *Anaeromyces*, *Buwchfawromyces,* and unclassified Neocallimastigaceae. Overall, transition of steers from a baseline hay diet to alfalfa pastures was associated with drastic changes in the composition of the fungal community, but the overall composition of ARF did not differ (*p* > 0.05) among bloated and non-bloated steers. A correlation network analysis of the proportion of ARF and ruminal bacterial communities identified hub fungal species that were negatively correlated with several bacterial species, suggesting the presence of inter-kingdom competition among these rumen microorganisms. Interestingly, the number of negative correlations among ARF and bacteria decreased with frothy bloat, indicating a potential disruption of normal microbial profiles within a bloated rumen ecosystem. A better understanding of fungal-bacterial interactions that differ among bloated and non-bloated rumen ecosystem could advance our understanding of the etiology of frothy bloat.

## 1. Introduction

Alfalfa (*Medicago sativa* L.) is a perennial legume with the potential to improve productivity and sustainability of pasture-based beef production [1]. However, susceptibility of cattle to frothy bloat, a common digestive disorder associated with grazing of vegetative alfalfa can cause mortalities [2] and has hampered the widespread inclusion of this forage in grazing systems. Among the many factors identified to contribute to the development of frothy bloat, the disruption of rumen microbial fermentation appears to be of central importance [2]. In a functioning rumen, interactions among different species of bacteria, fungi, archaea, and protozoa results in the sequential breakdown and fermentation of non-structural and structural plant polysaccharides [3]. However, the high concentration of fermentable proteins and carbohydrates in vegetative alfalfa can promote the unrestrained proliferation of bacteria within the rumen. These bacteria in turn produce excessive amounts of exopolysaccharides (i.e., slime) and fermentation end products, increasing the viscosity of rumen fluid and generating a stable foam that traps gas and prevents it from being expelled by eructation [2,4]. Several mitigation strategies have been developed to control the incidence of frothy bloat in alfalfa grazing systems [2]. These include selection for bloat-resistant alfalfa cultivars (e.g., AC Grazeland [5]), the use of water-soluble pluronic detergents that reduce the viscosity of rumen contents [6], and incorporation of non-bloating forages containing condensed tannins into alfalfa pastures [7]. Although plant factors and physiological mechanisms underlying the development of frothy bloat are reasonably well-described, contribution of the different groups of ruminal microorganisms to bloat and how they may be manipulated to prevent it are poorly understood. 

Changes in the rumen bacterial communities during alfalfa-induced frothy bloat and in response to bloat preventatives have been previously described [8]. Anaerobic rumen fungi (ARF) also play an important role in sequential breakdown and fermentation of plant polysaccharides. These strictly anaerobic microorganisms are known as primary colonizers of fibrous plant materials [9]. Upon attachment to plant cell walls, the zoospores of ARF produce rhizoidal structures capable of penetrating the lignocellulosic tissues of the plant cell wall, thereby providing access to fermentable carbohydrates within the cell interior that are not readily accessible by rumen bacteria [10,11]. In addition to physical deconstruction of the plant cell wall, ARF also possess a wide variety of polysaccharide degrading enzymes, including cellulases, hemicellulases, and xylanases that contribute to the enzymatic hydrolysis of plant polysaccharides [9,12]. Due to this diverse enzymatic repertoire, interspecies interactions and nutrient competition between ARF and rumen bacteria have been the subject of several in vitro studies [13,14,15]. However, given the immense diversity and complexity of rumen microbiota, in vitro assays fail to provide a comprehensive picture of microbe-microbe interactions within the rumen ecosystem. Alternatively, correlation network analysis has emerged as a promising tool for exploring co-occurrence patterns within complex microbial communities, providing insights into potential interspecies interactions and microbial community dynamics within the rumen ecosystem [16,17]. 

Currently, ARF are classified into 18 distinct genera belonging to the phylum Neocallimastigomycota [18]. Among these, *Neocallimastix*, *Piromyces*, *Caecomyces*, *Cyllamyces*, *Orpinomyces*, *Anaeromyces, Pecoramyces,* and *Buwchfawromyces* have been previously identified in the rumen and fecal contents of cattle using a combination of culture-dependent and marker-gene sequencing methodologies [19,20,21,22,23]. These genera can be distinguished by morphological features, or through genetic variations in the internal transcribed spacer (ITS) region of the ribosomal RNA (rRNA) locus [24,25]. In the present study, we hypothesized that the interrelationships between anaerobic fungi and bacterial communities would be indicative of a dysbiosis in the rumen of bloated cattle. We further investigated if the administration of the pluronic detergent, Alfasure^®^ in water and inclusion of condensed tannin containing sainfoin would preclude the development of this dysbiotic state in cattle grazing alfalfa pastures. 

## 2. Materials and Methods 

### 2.1. Ethics Statement 

Protocol (1214) for this experiment was reviewed in April 2012 and approved by the Animal Care Committee of the Lethbridge Research and Development Centre (LRDC), Agriculture and Agri-Food Canada, Lethbridge, Alberta. Cattle were cared for according to the guidelines of the Canadian Council on Animal Care [26].

### 2.2. Experimental Design, Animal Management, and Assessment of Bloat Scores 

Twelve ruminally-fistulated Aberdeen Angus steers were allocated to a 3 × 3 crossover experiment, subjecting all animals to three different treatments evenly distributed across three 11-day time periods. An adaptation period (baseline phase) of 3 weeks preceded the experiment during which all steers were offered ad libitum alfalfa hay. Steers were then subjected to three different treatments: (1) pure alfalfa pasture (PA), (2) pure alfalfa pasture with Alfasure (AA), and (3) mixed alfalfa—sainfoin pasture (AS). During both PA and AA treatments, steers grazed pure stands of alfalfa, but steers in AA treatment additionally received the pluronic detergent Alfasure (0.25 mL/L) in their drinking water to prevent bloat. This dosage delivered ≈ 9.5 g/d of poloxalene to each animal. During the AS treatment, steers grazed alfalfa pasture containing sainfoin which accounted for ≈ 15% of pasture dry matter (DM). Each experimental period consisted of 11 days; an adaptation/washout phase (7 days; during which confined steers were offered alfalfa hay ad libitum) and a sampling phase (4 days, during which steers were grazed on pastures). On each of the sampling days, all groups were released at 08:30 into either pure vegetative alfalfa (PA and AA treatments) or mixed vegetative alfalfa–sainfoin pastures (AS treatment). Stocking densities were adjusted by using electric fencing to ensure that pastures were always vegetative and steers were allowed to graze freely for 6 h until 14:30. Steers were then removed from the pasture and housed in a pen with freely available water, but no feed. 

Steers were monitored every 30 min during grazing and for 2 h after grazing to record incidences of bloat according to the protocol described by Majak et al. [27]. In summary, bloat scores were assigned as follows: 0  =  Normal (no visible sign of bloat), 1  =  Slight (slight distention of the left side of the animal), 2  =  Marked: (marked distention of the left side of the animal with an asymmetrical egg-shape as observed from the rear), and 3 = Severe (severe distention on the left side so that it is observable along the top of back from the right side of the animal). A single steer bloating on one day was counted as single bloat incidence. In the PA treatment, once the sum of bloat incidences reached 9 or more over the sampling period, steers were crossed over between treatments and the period was repeated until an additional 9 cases of bloat occurred on the PA treatment. 

### 2.3. Rumen Sample Collection and Processing

For the purpose of this study, rumen samples collected from steers that did not bloat (score of 0) were compared to those from steers that achieved a bloat score of 1 to 3. Baseline samples of rumen digesta were collected from each steer at the end of the primary adaptation period. All rumen samples consisted of composites collected from the cranial, caudal, and ventral sacs. In all treatment periods, after the steers were introduced to the pasture, rumen digesta were collected between days 8 and 11, immediately after they were removed from their assigned paddock in the afternoon. 

Approximately 50 g of total ruminal digesta was collected and transferred into a heavy-walled 250-mL beaker and squeezed with a Bodum coffee maker plunger (Bodum Inc., Triengen, Switzerland). Solid residue was suspended in 30 mL of cold (4 °C) grinding buffer (100 mM Tris-HCl, 500 mM EDTA, 1.5 M NaCl, 1 mL proteinase K, pH 8.0). The extruded liquid was placed in a shallow aluminum foil dish and flash frozen in liquid nitrogen. All samples were stored at −80 °C.

### 2.4. DNA Extraction and Quality 

For the purpose of this study, frozen solid residues were ground under liquid nitrogen in a precooled porcelain mortar. Samples were then transferred into a precooled Retsch RM 100 Mortar Grinder equipped with a stainless-steel mortar bowl and pestle (F. Kurt Retsch GmbH and Co. KG, Haan, Germany) and ground for a further 5 min under liquid nitrogen. As needed, liquid nitrogen was added to the mortar bowl during grinding to maintain the mixture in a semi-fluid state. Genomic DNA was then extracted from 150 to 250 mg of each sample using QIAamp DNA Stool Mini Kit following the manufacturer’s protocol (Qiagen Inc., Mississauga, ON, Canada). 

### 2.5. Library Construction and Illumina Sequencing

For bacterial communities, sequencing libraries targeting the V3-V4 region of the 16S rRNA gene were constructed as described previously [8]. For preparation of fungal sequencing libraries, the fungal-specific primer set of the Earth Microbiome Project, ITS1f (5′-CTTGGTCATTTAGAGGAAGTAA-3′) and ITS2 (5′-GCTGCGTTCTTCATCGATGC-3′) was used to target the internal transcribed spacer region of the fungal genomes [28]. PCR reaction for each sample was performed in duplicate and contained 1.0 µL of pre-normalized DNA (20 ng/µL), 1.0 µL of each forward and reverse primer (10 µM), 12 µL of molecular grade water (Fisher Scientific, Ottawa, ON, Canada) and 10 µL of 5 Prime Hot MasterMix (5 Prime Inc., Gaithersburg, MD, USA). Reactions consisted of an initial denaturing step at 94 °C for 2 min followed by 35 amplification cycles at 94 °C for 45 s, 52 °C for 45 s, and 70 °C for 60 s; finalized by an extension step at 70 °C for 10 min in an Eppendorf Mastercycler pro (Eppendorf, Hamburg, Germany). PCR products were then purified using ZR-96 DNA Clean-up Kit (ZYMO Research, Irvine, CA, USA) to remove primers, dNTPs and reaction components. Fungal ITS2 libraries were then generated by pooling 200 ng of each sample as quantified by Picogreen dsDNA (Invitrogen, Burlington, ON, Canada). This was followed by multiple dilution steps using pre-chilled hybridization buffer (Illumina, San Diego, CA, USA) to bring the pooled amplicons to a final concentration of 5 pM, as measured by a Qubit 2.0 Fluorometer (Life technologies, Burlington, ON, Canada). Finally, 15% of PhiX control library was spiked into the amplicon pool to improve the unbalanced and biased base composition of the fungal ITS libraries. Customized primers for sequencing reads R-1 (5′-TTGGTCATTTAGAGGAAGTAAAAGTCGTAACAAGGTTTCC-3′), R-2 (5′-CGTTCTTCATCGATGCVAGARCCAAGAGATC-3′), and R-Index (5′-TCTC GCATCGATGAAGAACGCAGCCG-3′) were synthesized and purified by polyacrylamide gel electrophoresis (Integrated DNA Technologies, Coralville, IA, USA) and added to the MiSeq Reagent Kit V3 (600-cycle) (Illumina, San Diego, CA, USA). The 300 paired-end sequencing reaction was performed on a MiSeq platform (Illumina, San Diego, CA, USA) at the Department of Animal Science, University of Manitoba, Canada. Bacterial and fungal sequencing data were uploaded into the Sequence Read Archive (SRA) of NCBI (http://www.ncbi.nlm.nih.gov/sra) and accessible through accession number PRJNA663462. Metadata used for bioinformatics and statistical analyses of both bacterial and fungal communities are in Supplementary File S3.

### 2.6. Bioinformatics 

The default settings of FLASH assembler (version 1.2.11 [29]) were used to merge the overlapping paired-end Illumina fastq files. UPARSE algorithm (version 9 [30]) was used for quality filtering of the reads based on maximum expected error value = 1.0 (using options “-fastq_filter” and “-fastq_maxee 1”). The UNOISE2 algorithm [31] was then used to further refine sequencing reads and generate amplicon sequence variants (ASVs). For bacterial ASVs, taxonomies were assigned by UCLUST consensus taxonomy assigner (version = 1.2.22) [32] using the GreenGenes database (release May 2013). For fungal ASVs, taxonomies were assigned by SortMeRNA classifier (version 2.0, 29/11/2014) [33] using the UNITE dynamic database (version 8, 04/02/2020 [34]). Unassigned ASVs, and those assigned to the class of Chloroplast and family of Mitochondria were removed from the final bacterial ASV table. Unless specified otherwise, all samples were rarefied to 25,000 bacterial ASV/sample for downstream analyses. Due to the importance of anaerobic rumen fungi in fiber degradation and ruminal fermentation, the fungal ASV table was filtered so that only reads belonging to the Neocallimastigomycota (strict anaerobe rumen fungi) were retained. The subsequent fungal ASV table was rarefied to 7000 ASV/sample for downstream analyses.

### 2.7. Statistical Analysis

Differences in Shannon index (diversity) and Chao1 index (richness) of rumen fungi between the baseline hay diet (*n* = 12) and each treatment diet (PA, *n* = 11; AA, *n* = 10; and AS, *n* = 11) were assessed using the Wilcoxon signed-rank test for matched-pairs. Diversity and richness were also compared between steers that developed bloat (*n* = 6) and those that did not (*n* = 5) while grazing alfalfa-sainfoin pastures.

Microbiota present in at least 50% of samples were used to assess rumen fungi (79 ASVs) and bacterial composition (1518 ASVs). Redundancy analyses (Vegan package) [35] were performed to assess the observed variation in the composition of rumen fungi explained by diet (overall). For each treatment, diet was compared to the baseline and bloat-status within the alfalfa–sainfoin diet. Identical analyses were performed to assess the variation in rumen bacterial composition explained by diet and bloat-status. ASV counts were center-log transformed using the CoDaSeq package [36] after imputation of zeros using a Bayesian multiplicative replacement method implemented in the zCompositions package [37].

Correlation network analysis (CoNet [38]) was used to assess co-occurrence and mutual exclusion relationships between bacterial and fungal ASVs and identify fungal hub ASVs that showed the highest number of positive/negative correlations within bacterial communities. In CoNet’s ensemble method, a combination of different correlation and dissimilarity measures (Spearman’s rank correlation coefficient, Kendal correlation coefficient, Bray–Curtis dissimilarity distance, and Kullback–Leibler Divergence) were used to infer co-occurrence networks. In brief, for each measure, distributions of all pairwise scores between the nodes (a node representing the relative abundance of an ASV that was found in at least 50% of the samples) were computed. For each measure and edge (an edge representing a positive or negative correlation between two nodes), 1000 permutations were conducted, including a renormalization step for Spearman correlations in order to address the issue of compositionality introduced by different sequencing depths among samples. For all correlation and dissimilarity measures, *p* values were computed as the probability of the null value (represented by the mean of the null distribution) under a Gauss curve generated from the mean and standard deviation of the bootstrap distribution. Measure-specific *p* values were then merged using Brown’s method [39] and subjected to Benjamini–Hochberg’s FDR correction. An edge was considered significant and kept in the final network if (a) it was supported by at least three measures, (b) it had a merged *p* value below 0.05, and (c) it was within the 95% confidence interval defined by the bootstrap distribution. Using the above methodology, an analysis was conducted including all steers with both bacterial and fungal data (the overall network), as well as a stratified analysis to assess the potential for unique interactions within those steers that bloated and those that did not. The 4 ASVs that had the most connecting edges (most interactions) within the overall network, within the no-bloat network and/or within the bloat network (10 ASVs in total) were further assessed as potentially important ASVs (i.e., hub ASVs). A second redundancy analysis was performed to assess observed variation in rumen bacterial communities explained by the selected fungal “hub ASVs”. The observed variation in overall bacterial composition explained by each fungal hub ASV was assessed, along with the total variation explained by all selected hub ASVs (using an R^2^ adjusted for multivariate analysis). Total variation explained by hub ASVs was also assessed within bloated and non-bloated steers. 

An analysis of variance permutation test (lmPerm package) [40], with steer as a random factor was used to associate diet and bloat status with center-log transformed ASV abundances (for 133 ASVs present in at least 10% of samples). Correction of *p*-values across ASVs was done using the Benjamini–Hochberg procedure. Pairwise comparisons were performed for the 51 ASVs identified as being significantly associated with diet/bloat-status combination after *p*-value correction (P_FDR_ < 0.05). Specifically, the baseline diet was compared to other diet/bloat-status combinations AA (no bloat, *n* = 10); PA (bloat, *n* = 11); AS (bloat, *n* = 6); and AS (no bloat, *n* = 5) using the Wilcoxon signed-rank test for matched pairs and *p*-values were corrected across ASVs (within each test) using the Benjamini–Hochberg procedure. Further, to assess the effect of bloat within diet, bloat status was compared within the alfalfa–sainfoin diet using the same *p*-value correction method. 

## 3. Results

### 3.1. Bloat Incidence 

Grazing steers on PA pasture resulted in 11 out of 12 steers developing frothy bloat over the course of the three periods, with one steer being removed from the trial after the first period due to lameness. Conversely, the addition of Alfasure^®^ to drinking water completely prevented bloat in all steers across all treatment periods. Grazing steers on AS pasture was moderately effective at preventing bloat, with 5 out of 11 steers not bloating on this treatment (Table 1).

### 3.2. Sequencing Results and Phylogenetic Diversity of the Fiber-Associated Microbial Communities

Taxonomic assignment of ASVs resulting from fungi-specific ITS primer set revealed the presence of three dominant fungal phyla across all samples, including Neocallimastigomycota 78.67% [SD (standard deviation) = 18.30%], Ascomycota 13.18% (SD = 14.57%), and Basidiomycota 1.85% (SD = 0.28%). The final ASV table was filtered so that only the Neocallimastigomycota were retained for downstream analysis. After quality filtering, the final ASV table for fiber-associated ARF contained an average of 111,724 (SD = 67,191) ASVs per sample, comprised of 189 unique ASVs. Within the Neocallimastigomycota, eight distinct genera were identified across all samples, including *Neocallimastix* 14.70% (SD = 8.20%), *Caecomyces* 28.06% (SD = 16.96%), *Orpinomyces* 18.00% (SD = 13.08%), *Piromyces* 21.11% (SD = 15.42%), unclassified Neocallimastigaceae 13.06% (SD = 12.73%), *Cyllamyces* 10.89% (SD = 14.64%), *Anaeromyces* 0.60% (SD = 0.59%) and *Buwchfawromyces* 0.45% (SD = 0.71%). The final ASV table for bacterial communities contained an average of 55,575 (SD = 18,676) reads per sample, comprised of 5554 unique ASVs. Taxonomic assignment of ASVs resulting from V3-V4 primer set revealed the presence of five dominant phyla including Bacteroidetes 54.27% (SD = 9.64%), Firmicutes 28.71% (SD = 6.94%), Fibrobacteres 6.89% (SD = 3.91%), Spirochaetes 4.09% (SD = 1.86%), and Proteobacteria 0.78% (SD = 0.34%). Detailed description of bacterial communities and their associations with bloat development and preventive strategies in this experiment have been previously described [8]. 

### 3.3. Diversity of Anaerobic Rumen Fungi as Impacted by Diet and Bloat-Status

**Figure 1:**
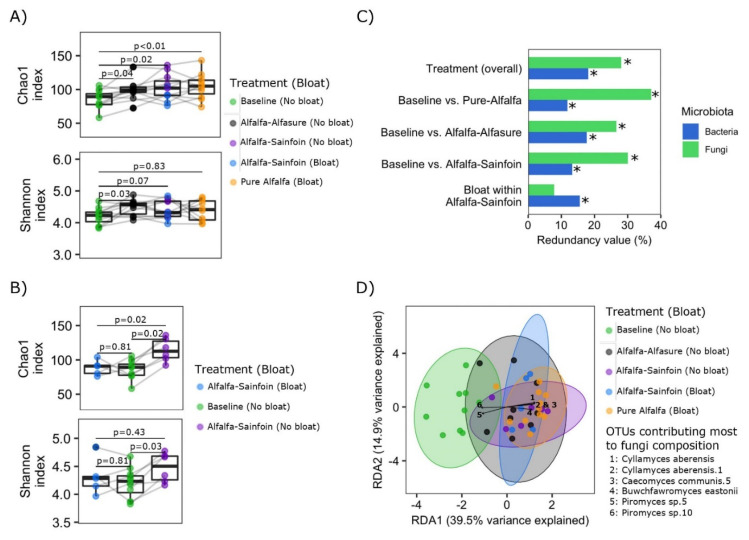
Comparisons of richness, diversity, and composition of rumen fungi communities in steers fed alfalfa hay or grazing alfalfa or alfalfa-sainfoin pastures. (**A**) Comparison of richness (Chao1 index) and diversity (Shannon index) among treatment groups and (**B**) comparisons of richness (Chao1 index) and diversity (Shannon index) among baseline hay diet and bloated and non-bloated steers on the alfalfa-sainfoin diet. Boxes show the interquartile range with the line at the median and whiskers showing minimum and maximum values. A line between two individual dots indicates the same steer in different groups. Comparisons were tested using Wilcoxon signed-rank test. (**C**) Summary statistics of redundancy analyses assessing the association of the overall composition of fungal and bacterial communities with treatments in general (top), individual pair-wise comparisons between baseline hay diet and each of the alfalfa pasture treatments (three middle), and bloated vs. non-bloated steers within the alfalfa-sainfoin treatment (bottom). Redundancy values indicate the percentage of observed variation in fungal composition explained by each factor (Treatment overall, *n* = 44; Pairwise comparisons: Baseline vs. Prue-Alfalfa, *n* = 22; Baseline vs. Alfalfa-Alfasure^®^, *n* = 20; Baseline vs. Alfalfa-Sainfoin, *n* = 22; Bloat vs. non-bloat in Alfalfa-Sainfoin, *n* = 11). * = *p* < 0.05. (**D**) Redundancy analysis bi-plot depicting differences in fungal composition between treatment groups/bloat status. Arrows indicate 7 amplicon sequence variants (ASVs) that accounted for most of the differences in fungal composition (e.g., Neocallimastigaceae sp7 and sp3 (arrows ID 1 and 2) tended to be enriched in the rumen contents of steers fed the baseline diet).

Fungal richness (Chao1 index) and diversity (Shannon index) of rumen contents were compared between the alfalfa hay and the grazing treatments (Figure 1A), as well as between steers which bloated and those that did not when grazing AS (Figure 1B). Overall, fungal richness was lowest in the baseline hay diet, and increased with the grazing of AA and AS pastures. The greatest increase (compared to baseline) was observed when cattle were grazing pure alfalfa. Meanwhile, compared to the baseline diet, diversity increased (*p* = 0.03) only in steers that did not bloat (i.e., baseline vs. AA, *p* = 0.03; and baseline vs. AS no-bloat, *p* = 0.03). Steers that did not bloat while grazing AS also had higher (*p* = 0.02) fungal richness compared to those that bloated (Figure 1B). Overall, fungal diversity did not correlate with the diversity of rumen bacteria (*rho* = 0.23, *p* = 0.15), even after stratification by bloat-status (No bloat, *rho* = 0.24, *p* = 0.26; Bloat, *rho* = 0.074, *p* = 0.78).

### 3.4. Composition of Anaerobic Rumen Fungi 

Overall, composition of rumen fungi was largely associated with diet (i.e., baseline hay diet vs. alfalfa pastures), which explained over 30% of the observed variation in the ARF composition (Figure 1C,D). Within the AS treatment, although development of bloat did not influence the overall composition of ARF, it did influence the bacterial composition [(*p* < 0.05); Figure 1C)]. ADONIS pairwise comparison also revealed considerable differences in the overall composition of ARF between the baseline hay diet and other treatments [baseline vs. PA, R^2^ = 0.44 and *p* < 0.01, baseline vs. AA, R^2^ = 0.36 and *p* < 0.01; baseline vs. AS (no-bloat) R^2^ = 0.30 and *p* < 0.01; baseline vs. AS (bloat) R^2^ = 0.35 and *p* < 0.01; Figure S1]. Certain ASVs were found to make the largest contribution to differences in the composition of ARF between treatments; *Piromyces* sp.5 and *Piromyces* sp.10 were associated with the baseline diet whereas in general, *Cyllamyces aberensis*, *Cyllamyces aberensis*.1, *Caecomyces communis.5*, and *Buwchfawromyces eastonii* were associated with steers grazing alfalfa (Figure 1D). 

**Figure 2:**
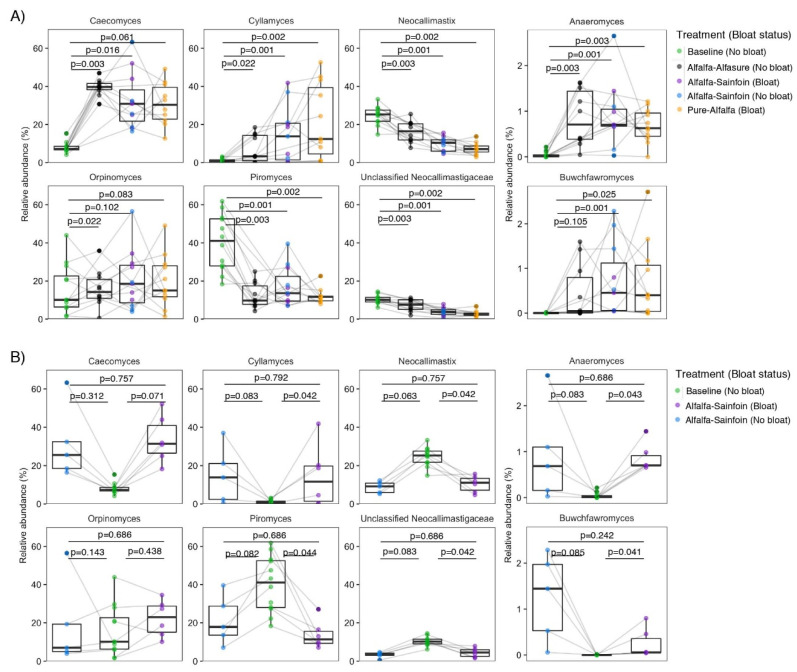
Pair-wise comparisons of the proportion of anaerobic rumen fungal genera (**A**) between baseline and each of the treatments, and (**B**) among steers that did and did not bloat while grazing alfalfa-sainfoin and the baseline alfalfa hay diet. Boxes show the interquartile range with a line at the median and whiskers showing minimum and maximum values. A line between two individual dots indicates the same steer in different groups. Comparisons were tested using Wilcoxon signed-rank test on center log-ratio proportions. FDR corrected *p*-values were obtained using the Benjamini–Hochberg procedure. *p*-values for all possible pair-wise comparisons can be found in Supplementary File S1.

The relative proportions of fungal genera were altered following transition from baseline diet to alfalfa pastures (Figure 2A). We observed that regardless of bloat incidence or inclusion of sainfoin or Alfasure^®^, transition from the baseline diet to alfalfa pastures enriched *Cyllamyces, Caecomyces, Anaeromyces,* and *Buwchfawromyces*; whereas, *Neocallimastix*, *Piromyces,* and unclassified Neocallimastigaceae accounted for a larger proportion of the fungal population in the rumen of steers consuming the baseline diet. Compared to other genera, *Orpinomyces* appeared to be more universally distributed among steers regardless of treatment. Among all possible pair-wise comparisons, the proportion of *Neocallimastix*, *Piromyces,* and unclassified Neocallimastigaceae were significantly lower in bloated steers on AS compared to the baseline diet (Figure 2B; also see Supplementary File S1 for Benjamini-Hochberg corrected *p*-values of all pair-wise comparisons of genera among treatments). 

### 3.5. Co-Occurrence Patterns of Anaerobic Rumen Fungi with the Rumen Bacterial Community in Relation to Treatments

**Figure 3:**
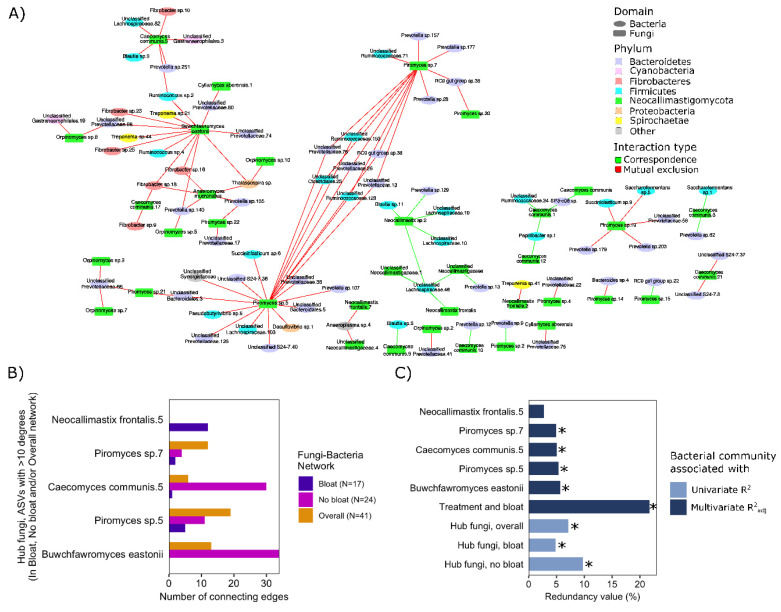
Co-occurrence patterns between anaerobic rumen fungi and bacteria and association of hub fungal ASVs with bacterial composition. (**A**) The overall network showing associations between fungi and bacteria among all steers and across all treatments (separate networks showing fungal-bacterial associations in bloated and non-bloated steers can be found in Supplementary Figure S2A,B, respectively). Nodes (representative of ASVs) are colored based on originating phyla, and edges (representing significant co-occurrence/co-exclusion relationships) are colored based on the type of relationships (red = negative relationship or mutual co-exclusion and green = positive relationship or co-occurrence). Only edges showing significant associations (P_(FDR)_ < 0.05) supported by at least three measures of correlation and/or dissimilarity are included in the network (Spearman rank correlation, Kendal correlation, Bray–Curtis and Kullback–Leibler dissimilarity). (**B**) Potential hub fungi ASVs identified across all networks (non-bloated steers, bloated steers and overall networks). The number of connecting edges indicates the number of associations each fungi ASV has with bacterial ASVs in each network. The y-axis shows ASVs with the most connecting edges (potential hub fungi) for each of the three networks. (**C**) Redundancy analyses showing the contribution of hub fungi ASVs to observed variation in overall bacterial composition (Redundancy value using center log-ratio proportions of fungal ASVs). Individual hub fungi were assessed in a single redundancy model adjusted for dietary treatment and bloat status (top six bars colored in dark blue). Three additional models (bottom three bars colored in light blue) show the combined contribution of all 10 hub fungi to observed variations in overall bacterial composition of the rumen content of all steers (Hub fungi, overall), overall bacterial composition of the rumen content of non-bloated steers (Hub fungi, no bloat), and overall bacterial composition of the rumen content of bloated steers (Hub fungi, bloat). * indicates *p* < 0.05.

Co-occurrence patterns between fungal and bacterial ASVs were assessed across all steers (Figure 3A), as well as between steers that did and did not bloat (Figure S2A,B, respectively). Overall, a large percentage of observed interrelationships among fungal and bacterial ASVs were mutually exclusive (i.e., negative relationship between the proportion of two ASVs; including 81% of relationships in the overall network, 88% in bloat network, and 77% in non-bloated network), suggestive of the presence of competitive interactions among fungi and bacteria within the rumen. Within each network, the top 4 ASVs having the highest number of connections (i.e., significant relationships) with other ASVs in the network were all fungi. Figure S2B depicts fungal hub ASVs with a considerable number of connections to various rumen bacteria (showing > 10 significant +/− relationships in one network). These fungal hub ASVs were next assessed in downstream analyses for their potential influence on the composition of the rumen bacterial population (Figure 3A). Interestingly, redundancy analysis revealed that *Buwchfawromyces eastonii,* a major hub-ASV identified in the network analysis of steers without bloat, had the highest explanatory power regarding variations in ruminal bacterial composition (Figure 3B). Similarly, *Piromyces* sp. 5, *Caecomyces communis* 5, and *Piromyces* sp. 7, identified as hub-ASVs by network analysis of non-bloated steers, could also explain more than 5% of the overall variation in ruminal bacterial composition. The only major fungi hub-ASV in the network of steers with bloat (*Neocallimastix frontalis.* 5), generally did not explain as much variation in rumen bacterial composition (Figure 3B). Assessments of the combined influence of hub-fungi on ruminal bacterial composition revealed that changes in the proportion of hub-fungi were associated with bacterial composition in rumen contents from both non-bloated (R^2^_adj_ = 9.7%, *p* < 0.01) and bloated steers (R^2^_adj_ = 4.9%, *p* < 0.01; Figure 3C).

The proportions of ARF identified as hub ASVs also differed between the baseline hay diet and other treatment groups, as well as among steers that did and did not bloat while grazing AS (Figure S3A,B and Supplementary File S2). Compared to the baseline diet, the proportions of hub ASVs belonging to *Caecomyces communis*, *Cyllamyces aberensis*, *Anaeromyces,* and *Buwchfawromyces eastonii* increased in the rumen contents from steers grazing alfalfa pastures. Among hub ASVs, *Buwchfawromyces eastonii* was particularly interesting, being among the ASVs that contributed most to the difference in fungal composition between samples (Figure 1D), as well as being a hub ASV with over 30 connections to bacterial ASVs. The proportion of this fungi ASV increased when steers were switched from the baseline diet to alfalfa pastures. However, the proportion of this ASV was lower in the rumen content of steers that bloated on the AS treatment compared to the ones that did not (Figure S3B). Although after FDR correction, no ASVs differed significantly between bloated and non-bloated steers on AS treatment, likely in-part due to the low sample size of this comparison (*n* = 6 bloat vs. 5 non-bloat). Before FDR correction, *Buwchfawromyces eastonii* was found to be enriched in the rumen content of steers that did not bloat on the AS treatment (*p* = 0.03, Supplementary File S2).

## 4. Discussion

The transition from alfalfa hay to alfalfa pastures and subsequent development of frothy bloat has previously been associated with the perturbation of rumen bacterial communities [8], but its impact on rumen fungal communities has not been investigated. In the current study, we characterized the fungal communities of the solid fraction of rumen digesta and their relationship to bacterial communities. In doing so, we provided a comprehensive description of the changes in the composition of anaerobic fungi during development of frothy bloat and characterized their co-occurrence patterns with rumen bacterial communities. In particular, we identified fungal hub species that were negatively correlated with a number of rumen bacterial species, suggestive of competitive interactions among these two major groups of ruminal microorganisms. We further observed that transition from alfalfa hay to alfalfa pasture, and subsequent development of frothy bloat was associated with drastic changes in the composition of ARF, but only exhibited moderate changes with the use of pluronic detergent or condensed tannin containing forages as preventatives.

Anaerobic rumen fungi are a phylogenetically unique group within the phylum Neocallimastigomycota. In the present study, the majority of sequencing reads obtained using the fungi-specific primer pair belonged to the phylum Neocallimastigomycota (~78%), enabling us to provide a comprehensive description of the ARF community within the rumen ecosystem. It is noteworthy to mention that our sequencing protocol was also able to detect aerobic fungal species belonging to the phyla Ascomycota and Basidiomycota. These fungi may represent epiphytic fungal populations inhabiting forages and other feed components, and likely do not contribute directly to ruminal fermentation. However, in a recent study utilizing metatranscriptomic data to extract 18S rRNA genes [41], authors also detected members of the phyla Ascomycota and Basidiomycota in the rumen contents of cattle, and suggested that these fungi may play an active role in the scavenging of oxygen that enters the rumen during feed and water consumption. We identified eight distinct genera of the phylum Neocallimastigomycetes, including *Neocallimastix, Caecomyces, Piromyces, Anaeromyces, Orpinomyces, Cyllamyces*, and *Buwchfawromyces* in over 80% of the ruminal samples collected from steers on both baseline and alfalfa pasture. Studies using ITS1 primer sets different than the one used in the present study have reported recovery of less than 23% of the sequencing reads assigned to ARF [19] and identified fewer ARF genera, including *Piromyces, Anaeromyces, Cyllamyces, Neocallimastix*, *Caecomyces,* and *Orpionmyces* [19,42]. By using a similar primer set to our study, Kumar et al. [22] reported recovery of around 1% of the sequencing reads belonging to ARF and identified three genera within this phylum, including *Cyllamyces, Caecomyces,* and *Orpinomyces*. Given the similarity of the primer set used to amplify fungal ITS regions, differences observed in the coverage of ARF between the latter study and ours could be mainly explained by differences in the fraction of the rumen digesta that had been used for isolation of metagenomic DNA. In our study, we isolated DNA from the solid (i.e., fibrous) fraction of the rumen digesta collected via the rumen cannula, whereas Kumar et al. [22] isolated DNA from rumen liquid collected via stomach tube. The main role of ARF within rumen ecosystem is to facilitate the decomposition of plant cell walls and enzymatic degradation of plant cell wall polysaccharides [10,11]. As such, the primary colonization site of ARF in the rumen ecosystem is believed to be fibrous plant materials [9], explaining why sequences belonging to ARF were enriched in our study. Moreover, differences in the taxonomic classifier used for fungal sequences could also contribute to differences in the coverage of ARF between the two studies—SortMeRNA classifier vs. RDP classifier [43]. In the present study, in addition to seven previously described genera of the phylum Neocallimastigomycota, we were also able to identify a group of unclassified ARF within the family Neocallimastigaceae. Previous studies have found evidence of uncharacterized isolates of rumen fungi, some of which may belong to the family Neocallimastigaceae [44,45]. 

Rumen fungi produce all the enzymes necessary for the degradation of plant material, and this enzymatic degradation may also be aided by mechanical deconstruction of plant cell walls as a result of the penetration of fungal rhizoids [46,47]. Previous in vitro research has shown that rumen fungi more readily degrade recalcitrant plant cell walls as compared to rumen bacteria, and that there is synergistic activity between fungi and bacteria in co-culture [48]. While fiber degradation is likely aided by both rumen bacteria and fungi, our network analysis suggests that the relationship between bacteria and fungi is generally competitive. Rumen bacteria and fungi also can compete for the same substrates (e.g., fiber) in the rumen. In support of this, our correlation network analysis showed many negative associations between rumen fungi and bacterial groups associated with fiber degradation, including members of Fibrobacteraceae, Ruminococcaceae, Christensenellaceae, and the genus *Treponema* [49,50]. Negative interactions between rumen fungi and cellulolytic bacterial species of *Ruminococcus* have been identified in previous in vitro studies [47,51]. This was hypothesized to involve the release of a polypeptides by ruminococci that inhibited cellulose hydrolysis by fungi. In our network analysis, rumen fungi also showed negative associations with *Prevotella,* a primarily amylolytic/proteolytic genus that is among the most abundant bacteria in the rumen [49]. The diverse enzymatic activity of rumen fungi also includes proteolytic and amylolytic activity [9,12,52], suggesting potential competition of this group of fungi with *Prevoltella* spp. for common substrates. Based on our results, two fungal species may be of particular importance: *Orpinomyces* (sp.8) and unclassified Neocallimastigaceae (sp.3) as they exhibited the most negative interaction with rumen bacterial species in cattle. In addition, they explained the largest amount of variation in overall bacterial composition of all the ten hub-fungi tested. 

The negative relationship between bacterial and fungal species observed in our network analysis was particularly notable in cattle that did not bloat; this relationship appeared to be disrupted in cattle that experienced bloat, with a large reduction in the number of negative associations between fungi and bacteria. In particular, hub fungal species showing the majority of negative relationships with bacteria in non-bloated rumen contents, were no longer associated with overall bacterial composition during bloat. The reduction in negative relationships between bacterial and fungal species following transition to alfalfa pasture and with the onset of bloat may be the result of reduced competition for substrates. As cattle were switched from the baseline hay diet to vegetative alfalfa pasture, increased availability of nutrients could provide a more permissive environment for competing species to proliferate at the same rate. Another possible explanation for the reduced negative correlations between fungi and bacteria could be the difference in the nature of rumen contents. During frothy bloat, rumen contents are enriched in bacterial slime, resulting from uncontrolled proliferation of certain bacterial populations. Thus, the slime-enriched rumen contents may not harbor a similar ratio of fungi:bacteria as that of normal rumen contents. Within the rumen of non-bloated cattle, a few positive associations were also identified between specific fungi and bacteria. These positive associations were primarily observed between *Neocallimastix* species and bacterial species within the genus *Treponema* and family Ruminococcaceae. *Treponema* spp. in the rumen are enriched by diets high in pectin, such as alfalfa hay [53,54]. Likewise, we found that the relative abundance of *Neocallimastix* was highest in alfalfa hay as the pre-grazing baseline diet. It is possible that *Treponema* and *Neocallimastix* thrive under similar dietary conditions, but in a non-competitive manner.

Condensed tannins within sainfoin are thought to be the primary components responsible for the bloat preventive properties of this forage, mainly as a result of a reduction in the rate of fermentation of soluble proteins and plant cell walls [55,56]. Sainfoin cultivars in our study possessed about 30 g of extractable condensed tannins per kg of forage DM [57]. Previous research has identified a reduction in the number of cellulolytic bacteria, such as *Ruminococcus* spp., with the inclusion of forages containing condensed tannins in the diet [58]. However, rumen fungi capable of degrading cellulose, such as *Neocallimastix patriciarum,* are thought to be less affected by condensed tannins than cellulolytic bacteria [59,60,61,62]. Of the fungi genera examined, only *Neocallimastix* was in a higher relative abundance in steers grazing alfalfa-sainfoin as compared to those grazing only alfalfa. Interestingly, in vitro, increases in pectin concentrations can reduce the extent to which condensed tannins limit the rate of cellulose digestion [53,54]. Our current research indicates positive interactions between pectinolytic bacteria (*Trepoonema* spp.) and *Neocallimastix* spp., though whether this is related to the interaction between pectin and tannins merits further investigation. 

The addition of sainfoin appeared to increase the diversity of fungal species compared to the baseline hay diet and grazed alfalfa, only in steers which did not bloat. This suggests a link between the bloat-prevention mechanism of sainfoin and rumen fungal diversity. High diversity has been suggested as a general indicator of a stable microbial community that is resistant to change during environmental perturbations [62], such as frothy bloat. However, the reason why inclusion of sainfoin increased the species diversity of rumen fungi in only a subset of animals in our study remains unknown. This may be in part explained by differences in selective grazing of sainfoin by individual animals. Whether reduced sainfoin intake in the subgroup of steers that developed bloat could be the reason for reduced fungal diversity, and potentially development of bloat, warrants further investigation. 

In the present study, transition from baseline hay diet to alfalfa pastures was associated with drastic changes in the composition of the fungal community. Based on PCoA of Bray–Curtis dissimilarity and RDA analysis, the composition of the fungal community during the baseline alfalfa hay diet differed from that observed in all other grazing treatments. However, the overall composition of the fungal community did not differ significantly among bloated and non-bloated steers on different treatments (i.e., PA, AA, and AS). This observation suggests that fungal populations were impacted more by the nature of the forage—i.e., whether the forage was conserved as hay or grazed—rather than the development of bloat. In contrast, our RDA analysis showed that a higher percentage of the variation in the composition of the ruminal bacterial community could be explained by the development of bloat. The observed differences in the response of these two groups of microorganisms to bloat could be attributed to their physiological characteristics. Bacterial populations can rapidly proliferate upon gaining access to readily fermentable carbohydrates and proteins within vegetative alfalfa, whereas the long life-cycle of anaerobic rumen fungi (8–32 h) [46] results in these microorganisms proliferating at a slower rate than bacteria. Furthermore, bacteria can produce excessive amounts of mucopolysaccharide “slime” that can interact with proteins to produce the stable froth that traps gases and results in bloat [2,4].

An important strength of the present study was our ability to comprehensively assess the overall composition of the ARF, including uncultivated/uncharacterized members of this group, and explore their response to bloat development and mitigation strategies. In particular, ITS gene sequencing of the solid fraction of rumen contents enabled us to recover a considerably higher percentage of the sequencing reads that belonged to ARF as compared to previous studies. Whereas, previous research on interactions between rumen fungi and bacteria has been mostly limited to in vitro microbe-microbe experiments, having access to both bacterial and fungal sequencing data enabled us to explore the global interrelationships among these two groups of microorganisms within the rumen ecosystem. However, care should be taken when interpreting the results of network analysis of amplicon sequencing data, as this type of analysis is prone to a high rate of false-positives [17]. Furthermore, while our experiment was carried out in a cross-over design that reduced inter-animal variability in the response of rumen microbiota (i.e., same steer receiving each of the treatments), a disadvantage of this approach is the potential carry-over effect between dietary treatments even after the 7-day adaptation phase that preceded the sampling period of each treatment. Another limitation of our study design that might also contribute to the development of bloat was the overnight feed deprivation prior to each grazing period, as this could affect both the grazing behavior of steers and the ruminal microbial profile. However, in this case this management strategy was deliberately employed to increase the likelihood of bloat. Nonetheless, being able to comprehensively describe the composition of anaerobic fungi in healthy and bloated rumen ecosystems, our results pave the way towards future research on the functional contribution of the rumen fungi to the development of frothy bloat. 

## 5. Conclusions

Our experiment provides novel insights into the dynamics of the rumen fungal community during adaptation to alfalfa pasture. In general, we observed that transition from baseline alfalfa hay diet to alfalfa pasture was associated with drastic changes in the diversity and composition of rumen fungal community, whereas the overall composition of this group of microorganisms was less affected by the development of bloat. In contrast, we observed that the ruminal bacterial community differed considerably between bloated and non-bloated steers, implying that rumen bacteria are the main drivers of frothy bloat. Rumen fungi play an important role in fiber degradation, and our results are in agreement with previous studies regarding general competition between rumen fungi and fiber degrading bacteria. Identification of novel fungal–bacterial interactions that differed among bloated and non-bloated rumen ecosystem merits further investigations to advance our understanding of the etiology of frothy bloat.

## Figures and Tables

**Table 1:** Summary of study design and incidences of bloat in grazing steers.

Period	Treatment ^1^					
	Pure Alfalfa		Alfalfa + Alfasure^®^		Alfalfa + Sainfoin	
	Bloat Incidence ^2^	Steers	Bloat Incidence	Steers	Bloat Incidence	Steers
Period 1	NB	0	NB	4	NB	0
	B	4	B	0	B	4
Period 2	NB	0	NB	3	NB	3
	B	3	B	0	B	1
Period 3	NB	0	NB	3	NB	2
	B	4	B	0	B	1

^1^ Within each period, steers were subjected to one of the three treatment groups including (1) pure alfalfa pasture (PA), (2) pure alfalfa pasture with Alfasure^®^ and (3) mixed alfalfa—15% sainfoin dry matter (DM) pastures. ^2^ Bloat incidence indicates the number of steers within each treatment groups that either bloated (B; bloat scores 1–3) or did not bloat (NB; bloat score 0). Each value indicates a single case of bloat in a single steer within a day.

## Supplementary Materials

The following are available online at https://www.mdpi.com/2076-2607/8/10/1543/s1, Figure S1: Beta-diversity of ruminal fungal communities. Figure S2: Co-occurrence patterns between fungi and bacteria in the solid rumen contents of (A) bloated and non-bloated (B) steers. Figure S3: Variations in the proportion of anaerobic rumen fungi amplicon sequence variants (A) bloated and non-bloated steers compared to those fed baseline diet (B) steers grazing alfalfa-sainfoin with and without bloat compared to baseline diet. File S1: Wilcoxon signed-rank tests. File S2: Amplicon sequence variants, baseline hay diet vs other treatments. File S3: Bacterial and fungal metadata.

## Abbreviations

AA	Alfalfa pasture with Alfasure^®^
ARF	Anaerobic rumen fungi
AS	Alfalfa—sainfoin
ASV	Amplicon sequence variants
B	Bloat
DM	Dry matter
ITS	Internal transcribed spacer
NB	no bloat
PA	Pure alfalfa pasture
rRNA	Ribosomal RNA
SRA	Sequence Read Archive
