# Supplementary material for: Interrelationships of Fiber-Associated Anaerobic Fungi and Bacterial Communities in the Rumen of Bloated Cattle Grazing Alfalfa
Source: Microorganisms. 2020 Oct 7;8(10):1543. doi: 10.3390/microorganisms8101543 (PMC7601590; doi:10.3390/microorganisms8101543)
Supplement: Supplementary file 1 [file microorganisms-08-01543-s001.zip › Figure_S1.pdf]

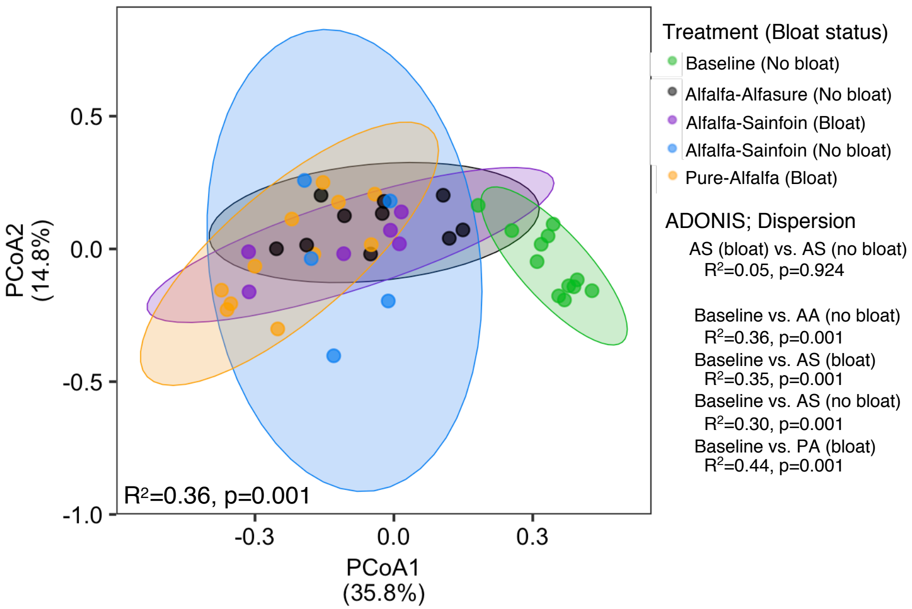

Figure S1. Beta-diversity of ruminal fungal communities. Principal coordinate analysis (PCoA) was used for visualization of Bray-Curtis dissimilarities of the fungal communities in the rumen content of steers subjected to different dietary regimens. Color codes were used to differentiate treatment groups and bloat status. The ADONIS package of R was used for performing Permutational Multivariate Analysis of Variance (PERMANOVA) to assess the effect of dietary treatments/bloat status on the overall composition of fungal communities. ADONIS R^2 for the overall model and pair-wise comparisons show the explanatory power of diets/bloat status to differentiate ruminal fungal communities. For all tests, p values < 0.05 were considered significant.
