# Supplementary figures and images for: Interrelationships of Fiber-Associated Anaerobic Fungi and Bacterial Communities in the Rumen of Bloated Cattle Grazing Alfalfa
Source: Microorganisms. 2020 Oct 7;8(10):1543. doi: 10.3390/microorganisms8101543 (PMC7601590; doi:10.3390/microorganisms8101543)

A)

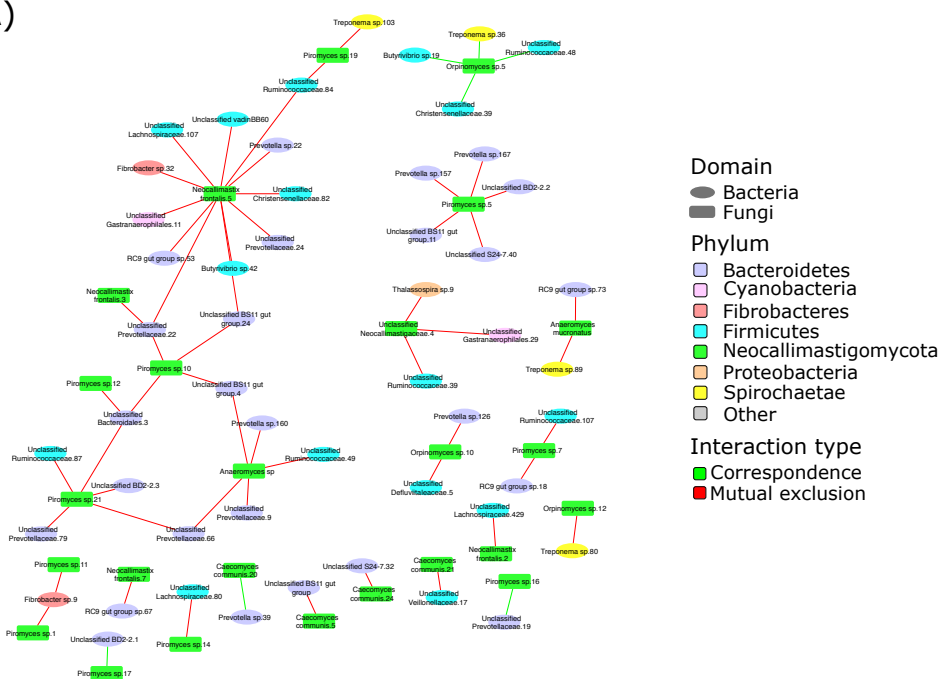

B)

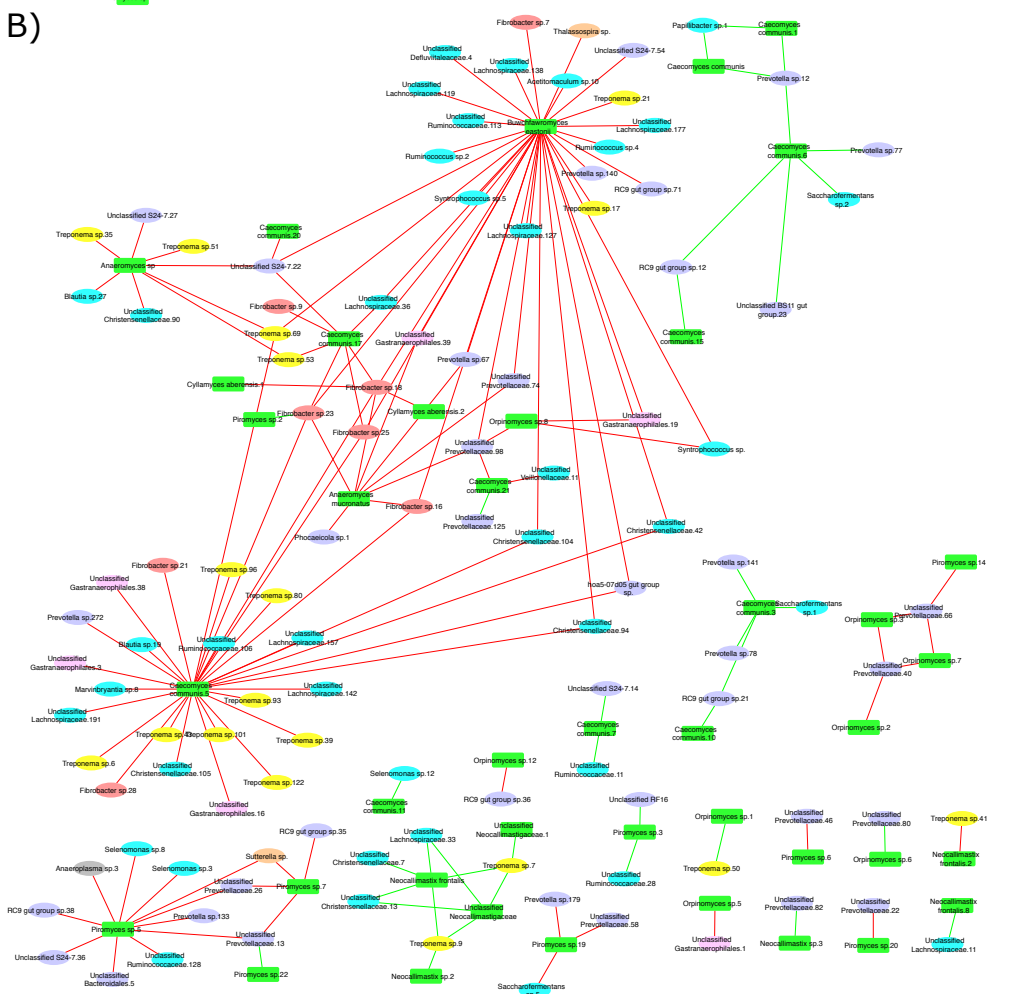

Supplement: Supplementary file 1 [file microorganisms-08-01543-s001.zip › Figure_S2.pdf]

A)

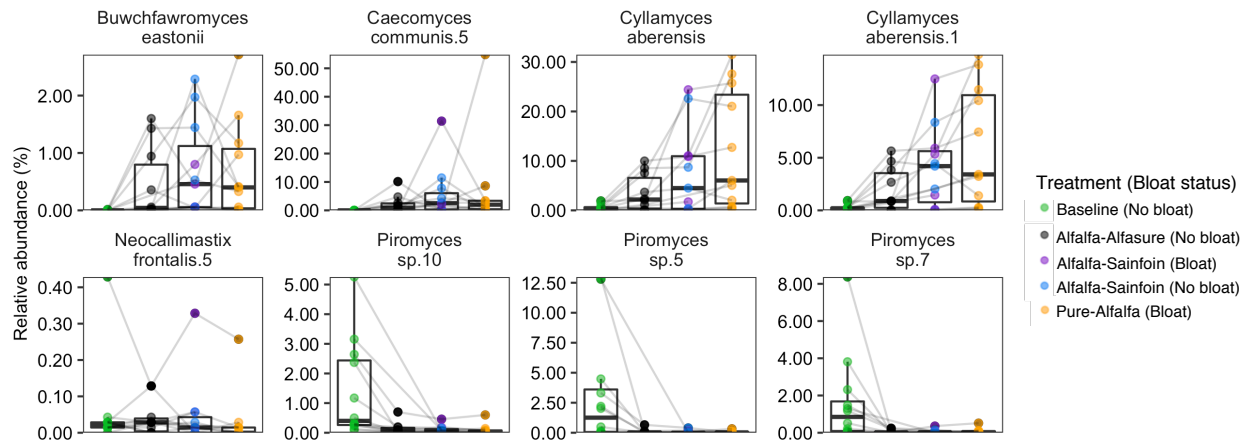

B)

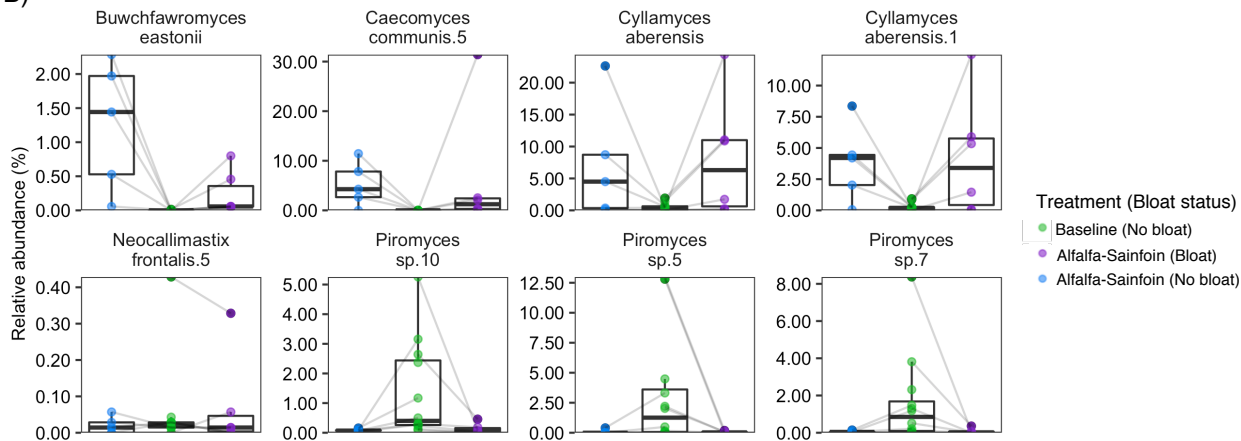

Supplement: Supplementary file 1 [file microorganisms-08-01543-s001.zip › Figure_S3.pdf]
